# Bis[tris­(1*H*-pyrazol-1-yl-κ*N* ^2^)methane]­nickel(II) bis­{[tris­(1*H*-pyrazol-1-yl-κ*N* ^2^)methane]­tris­(thio­cyanato-κ*N*)nickelate(II)} methanol disolvate

**DOI:** 10.1107/S1600536811045144

**Published:** 2011-11-05

**Authors:** Ganna Lyubartseva, Sean Parkin, Uma Prasad Mallik

**Affiliations:** a Department of Chemistry and Physics, Southern Arkansas University, Magnolia, AR 71753, USA; b Department of Chemistry, University of Kentucky, Lexington, KY 40506, USA

## Abstract

Attempts to prepare the mononuclear [(tpm)Ni^II^ *L*_3_]^−1^ [tpm = tris­(1*H*-pyrazol-1-yl)methane and *L* = thio­cyanate] anion yielded the methanol-solvated salt, [(tpm)_2_Ni^II^][(tpm)Ni^II^(NCS)_3_]_2_·2CH_3_OH or [Ni(C_10_H_10_N_6_)_2_][Ni(NCS)_3_(C_10_H_10_N_6_)]_2_·2CH_3_OH. The asymmetric unit consists of half a centrosymmetric bis­[tris­(1*H*-pyrazol-1-yl)methane]­nickel(II) cation and an octa­hedral nickelate(II) anion bound to one tpm and three *L* ligands, and a methanol solvent mol­ecule. One of the *L* ligands is disordered over two positions with occupancy factors of 0.650 (3) and 0.350 (3). There are O—H⋯S inter­actions between the methanol and the disordered thio­cyanate anion, and a weak C—H⋯O hydrogen bond between the cation and the methanol O atom.

## Related literature

For the ligand synthesis, see: Reger *et al.* (2000 ▶). For structural, spectroscopic and angular overlap studies of tris­(1*H*-pyrazol-1-yl)methane complexes, see: Astley *et al.* (1993 ▶). For background information on the modelling of metallo-enzyme sites by small mol­ecules, see: Kitajima *et al.* (1992 ▶); Trofimenko *et al.* (1992 ▶); Looney *et al.* (1992 ▶); Looney & Parkin (1994 ▶). A previous attempt to make similar building blocks with nickel(II) and a cyanide ligand is given in Lyubartseva & Parkin (2009 ▶). 
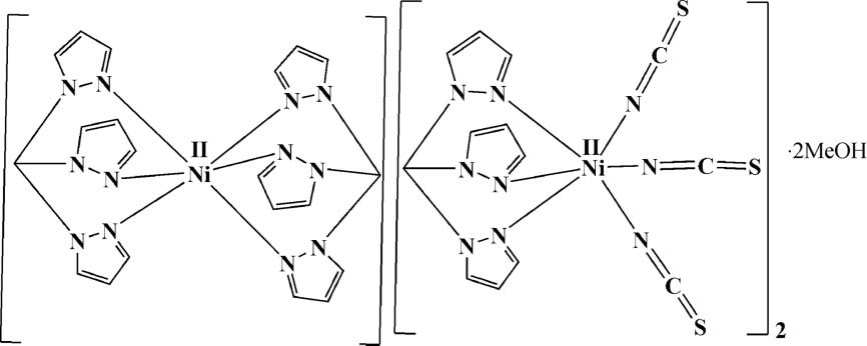


## Experimental

### 

#### Crystal data

[Ni(C_10_H_10_N_6_)_2_][Ni(NCS)_3_(C_10_H_10_N_6_)]_2_·2CH_4_O
*M* *_r_* = 1445.65
Monoclinic, 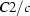

*a* = 33.4463 (8) Å
*b* = 7.3287 (2) Å
*c* = 27.2689 (7) Å
β = 112.590 (1)°
*V* = 6171.3 (3) Å^3^
*Z* = 4
Cu *K*α radiation
μ = 3.52 mm^−1^
*T* = 90 K
0.20 × 0.06 × 0.02 mm

#### Data collection

Bruker X8 Proteum diffractometer
Absorption correction: multi-scan (*SADABS* in *APEX2*; Bruker, 2006 ▶) *T*_min_ = 0.740, *T*_max_ = 0.933
41685 measured reflections
5605 independent reflections
4932 reflections with *I* > 2σ(*I*)
*R*_int_ = 0.061

#### Refinement

*R*[*F* ^2^ > 2σ(*F* ^2^)] = 0.049
*wR*(*F* ^2^) = 0.128
*S* = 1.11
5605 reflections
418 parameters
6 restraints
H-atom parameters constrained
Δρ_max_ = 1.34 e Å^−3^
Δρ_min_ = −0.44 e Å^−3^

### 

Data collection: *APEX2* (Bruker, 2006 ▶); cell refinement: *SAINT* (Bruker, 2006 ▶); data reduction: *SAINT*; program(s) used to solve structure: *SHELXS97* (Sheldrick, 2008 ▶); program(s) used to refine structure: *SHELXL97* (Sheldrick, 2008 ▶); molecular graphics: *XP* in *SHELXTL* (Sheldrick, 2008 ▶); software used to prepare material for publication: *SHELXL97* and local procedures.

## Supplementary Material

Crystal structure: contains datablock(s) global, I. DOI: 10.1107/S1600536811045144/ng5255sup1.cif

Structure factors: contains datablock(s) I. DOI: 10.1107/S1600536811045144/ng5255Isup2.hkl

Additional supplementary materials:  crystallographic information; 3D view; checkCIF report

## Figures and Tables

**Table 1:** Hydrogen-bond geometry (Å, °)

*D*—H⋯*A*	*D*—H	H⋯*A*	*D*⋯*A*	*D*—H⋯*A*
O1*S*—H1*S*⋯S3^i^	0.84	2.43	3.267 (4)	175
O1*S*—H1*S*⋯S3′^i^	0.84	2.88	3.459 (6)	128
C23—H23⋯O1*S*	1.00	2.15	3.118 (4)	162

Symmetry code: (i) 
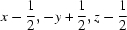
.

**Fig. 1.**
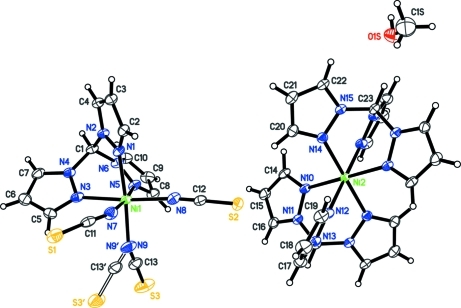
View of the title compound with displacement ellipsoids drawn at the 50% probability level. The minor isothiocynate disorder component is shown with open bonds. Unlabelled atoms are related to their labelled counterparts by inversion (0.5 - x, 1.5 - y, 1 - z).

**Fig. 2.**
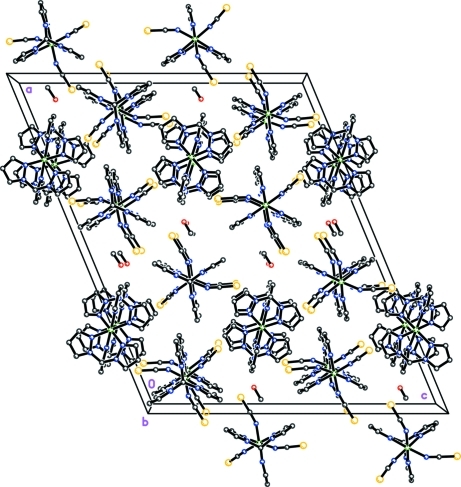
Packing diagram of the title compound as viewed down the b axis. The hydrogen atoms are omitted to enhance clarity.

## supplementary crystallographic information

### Comment

Tripodal ligands with three pyrazolyl groups are increasingly being used in small-molecule modeling of the active sites of metallo-enzymes in which the metal is coordinated to two or three imidazole groups from histidine (Kitajima *et al.* 1992, Trofimenko *et al.* 1992, Looney *et al.* 1992, Looney & Parkin 1994). One of the goals of this research is to explore the chemistry of the neutral ligand tris(pyrazol-1-yl)methane compared to the more extensively studied isoelectronic but anionic ligand tris(pyrazol-1-yl)borate. In attempts to prepare mononuclear [(tpm)Ni^II^*L*_3_]^-1^ , where tpm is tris(pyrazol-1-yl)methane, a symmetrical tripodal neutral nitrogen donor ligand, and *L* is NCS^-^, a uninegative N-donor pseudohalide anion, we obtained [(tpm)_2_Ni^II^][(tpm)Ni^II^(NCS)_3_]_2_.2CH_3_OH as blue monoclinic crystals in 57% isolated yield. The structure consists of centrosymmetric [bis[tris(1-pyrazolyl)methane-κ^3^]-nickel(II) cations, with Ni^II^—N distances ranging from 2.077 (2) to 2.082 (2) Å. The intraligand N—Ni—N angles in the cation range from 85.81 (10) to 95.27 (10)°, which introduces a slight trigonal distortion from perfect octahedral geometry. The anion consists of nickellate (II) atom surrounded octahedrally by one tripodal tris(pyrazol-1-yl)methane ligand and three isothiocyanate ligands. The tpm ligand N—Ni distances range from 2.080 (3) to 2.119 (3) Å, and the isothiocyanate N—Ni distances range from 2.046 (3) to 2.070 (3) Å.

### Experimental

The tris(pyrazolyl)methane ligand was synthesized according to the previously published procedure by Reger *et al.* (2000). Tetrabutylammonium thiocyanate was purchased from Aldrich and used as received. NiCl_2_.6H_2_0 (475 mg, 2 mmol) was dissolved in 15 ml methanol. Tris(pyrazolyl) methane (428 mg, 2 mmol) was dissolved in 15 ml methanol. The ligand solution was added dropwise to metal solution and with moderate stirring. Once the addition was complete, tetrabutylammonium thiocyanate (1.81 g, 6 mmol) was added. The solution was filtered and methanol was evaporated slowly. Blue crystals were obtained after 3 days (549 mg, 57% yield). Elemental analysis, calculated for Ni_3_C_48_H_48_N_30_S_6_O_2_ : C 39.88, H 3.35, N 29.07; found C 39.21, H 2.99, N 29.27%. IR (cm^-1^): 3361, 3133, 2977, 2071, 1516, 1440, 1400, 1284, 1247, 1220, 1088, 1050, 980, 905, 858, 788,  766, 660, 608, 475.

### Refinement

H atoms were found in difference Fourier maps and subsequently placed in idealized positions with constrained distances of 0.98 Å (RCH_3_), 1.00 Å (*R*_3_CH), 0.95 Å (C_sp2_H), 0.84 Å (O—H), and with *U*_iso_(H) values set to either 1.2*U*_eq_ or 1.5*U*_eq_ (RCH_3_, OH) of the attached atom.

To ensure satisfactory refinement of disordered parts of the structure, a combination of constraints and restraints were used. The *SHELXL97* constraints EXYZ and EADP were used to make the geometry and displacement parameters of closely proximate disordered atoms equal. The *SHELXL97* restraint command DELU was also used to ensure similar displacement parameters for closely proximate, chemically similar groups.

The final weighting scheme (SHELXL-97 command "WGHT"), which is optimized to give a flat analysis of variance, had a somewhat larger than usual value for the second parameter. This is generally attributed to some form of bias, such as could be caused by unrecognized twinning or some other kind of incomplete model. We observed no obvious cause for the unusual weighting scheme, but the available sample was far from perfect. Indeed, the crystals were covered in a blue powder, which was likely caused by partial drying of the crystal. Some of this blue powder was easy to remove, but some was stuck to the crystal surface and could not be removed without damaging the crystals.

In the final difference map there are small residual peaks clustered around the disordered isothiocyanate sulphur. This could perhaps be due to partial occupancy/disordered solvent, but all attempts to model it as such did not improve the refinement enough to warrant retention of the extra details.

### Crystal data

**Table:**

[Ni(C_10_H_10_N_6_)_2_][Ni(NCS)_3_(C_10_H_10_N_6_)]_2_·2CH_4_O	*F*(000) = 2968
*M**_r_* = 1445.65	*D*_x_ = 1.556 Mg m^−^^3^
Monoclinic, *C*2/*c*	Cu *K*α radiation, λ = 1.54178 Å
Hall symbol: -C 2yc	Cell parameters from 9957 reflections
*a* = 33.4463 (8) Å	θ = 2.9–67.8°
*b* = 7.3287 (2) Å	µ = 3.52 mm^−^^1^
*c* = 27.2689 (7) Å	*T* = 90 K
β = 112.590 (1)°	Rod, blue
*V* = 6171.3 (3) Å^3^	0.20 × 0.06 × 0.02 mm
*Z* = 4	

### Data collection

**Table:**

Bruker X8 Proteum diffractometer	5605 independent reflections
Radiation source: fine-focus rotating anode	4932 reflections with *I* > 2σ(*I*)
graded multilayer optics	*R*_int_ = 0.061
Detector resolution: 5.6 pixels mm^-1^	θ_max_ = 68.0°, θ_min_ = 2.9°
φ and ω scans	*h* = −39→39
Absorption correction: multi-scan (*SADABS* in *APEX2*; Bruker, 2006)	*k* = −8→8
*T*_min_ = 0.740, *T*_max_ = 0.933	*l* = −32→32
41685 measured reflections	

### Refinement

**Table:**

Refinement on *F*^2^	Primary atom site location: structure-invariant direct methods
Least-squares matrix: full	Secondary atom site location: difference Fourier map
*R*[*F*^2^ > 2σ(*F*^2^)] = 0.049	Hydrogen site location: inferred from neighbouring sites
*wR*(*F*^2^) = 0.128	H-atom parameters constrained
*S* = 1.11	*w* = 1/[σ^2^(*F*_o_^2^) + (0.0515*P*)^2^ + 28.6001*P*] where *P* = (*F*_o_^2^ + 2*F*_c_^2^)/3
5605 reflections	(Δ/σ)_max_ = 0.001
418 parameters	Δρ_max_ = 1.34 e Å^−^^3^
6 restraints	Δρ_min_ = −0.44 e Å^−^^3^

### Special details

**Table:**

Geometry. All e.s.d.'s (except the e.s.d. in the dihedral angle between two l.s. planes) are estimated using the full covariance matrix. The cell e.s.d.'s are taken into account individually in the estimation of e.s.d.'s in distances, angles and torsion angles; correlations between e.s.d.'s in cell parameters are only used when they are defined by crystal symmetry. An approximate (isotropic) treatment of cell e.s.d.'s is used for estimating e.s.d.'s involving l.s. planes.
Refinement. The crystals were covered in a blue powder, which was likely caused by partial drying of the crystal. Some of this blue stuff was easy to remove, but some was stuck to the crystal surface and could not be removed without damaging the crystal.In the final difference map there are small residual peaks clustered around the disordered thiocyanate group. This could perhaps be due to partial occupancy/disordered solvent, but all attempts to model it as such did not improve the refinement enough to warrant retention of the extra details.

### Fractional atomic coordinates and isotropic or equivalent isotropic displacement parameters (Å^2^)

**Table:**

	*x*	*y*	*z*	*U*_iso_*/*U*_eq_	Occ. (<1)
Ni1	0.388647 (18)	0.19922 (7)	0.82166 (2)	0.02134 (16)	
C1	0.38951 (11)	−0.2289 (4)	0.82452 (14)	0.0230 (7)	
H1	0.3895	−0.3653	0.8251	0.028*	
N1	0.33737 (9)	0.0180 (4)	0.80613 (11)	0.0212 (6)	
N2	0.34542 (9)	−0.1642 (4)	0.80957 (11)	0.0196 (6)	
C2	0.29596 (11)	0.0339 (5)	0.79732 (13)	0.0221 (7)	
H2	0.2810	0.1467	0.7933	0.027*	
C3	0.27681 (11)	−0.1379 (5)	0.79473 (13)	0.0236 (7)	
H3	0.2474	−0.1630	0.7885	0.028*	
C4	0.30942 (11)	−0.2617 (5)	0.80312 (13)	0.0214 (7)	
H4	0.3072	−0.3908	0.8042	0.026*	
N3	0.41898 (9)	0.0209 (4)	0.88658 (12)	0.0234 (6)	
N4	0.41572 (9)	−0.1623 (4)	0.87707 (11)	0.0220 (6)	
C5	0.44066 (11)	0.0375 (5)	0.93835 (14)	0.0253 (7)	
H5	0.4477	0.1506	0.9567	0.030*	
C6	0.45189 (12)	−0.1344 (5)	0.96261 (15)	0.0322 (8)	
H6	0.4677	−0.1592	0.9992	0.039*	
C7	0.43519 (11)	−0.2583 (5)	0.92251 (15)	0.0290 (8)	
H7	0.4369	−0.3874	0.9258	0.035*	
N5	0.41142 (9)	0.0136 (4)	0.77985 (12)	0.0241 (6)	
N6	0.40755 (9)	−0.1675 (4)	0.78705 (12)	0.0226 (6)	
C8	0.43166 (12)	0.0246 (5)	0.74650 (15)	0.0286 (8)	
H8	0.4389	0.1359	0.7340	0.034*	
C9	0.44093 (12)	−0.1481 (5)	0.73221 (15)	0.0322 (8)	
H9	0.4551	−0.1762	0.7089	0.039*	
C10	0.42528 (11)	−0.2688 (5)	0.75883 (15)	0.0286 (8)	
H10	0.4266	−0.3982	0.7578	0.034*	
N7	0.36703 (10)	0.3584 (4)	0.86862 (12)	0.0280 (7)	
C11	0.36862 (11)	0.3894 (4)	0.91106 (15)	0.0253 (8)	
S1	0.37247 (3)	0.43823 (13)	0.97092 (4)	0.0346 (2)	
N8	0.35651 (10)	0.3522 (4)	0.75539 (12)	0.0276 (6)	
C12	0.33809 (11)	0.4581 (5)	0.72297 (13)	0.0221 (7)	
S2	0.31218 (3)	0.61217 (12)	0.67928 (3)	0.0267 (2)	
N9	0.44458 (11)	0.3525 (4)	0.84076 (15)	0.0371 (8)	0.650 (3)
C13	0.4780 (2)	0.3745 (8)	0.8323 (3)	0.0346 (11)	0.650 (3)
S3	0.52288 (6)	0.4092 (2)	0.82170 (10)	0.0554 (6)	0.650 (3)
N9'	0.44458 (11)	0.3525 (4)	0.84076 (15)	0.0371 (8)	0.350 (3)
C13'	0.4749 (4)	0.3881 (14)	0.8716 (5)	0.0346 (11)	0.350 (3)
S3'	0.52609 (11)	0.4382 (5)	0.91591 (17)	0.0639 (14)	0.350 (3)
Ni2	0.2500	0.7500	0.5000	0.01688 (18)	
N10	0.30477 (8)	0.5976 (4)	0.54331 (10)	0.0187 (6)	
N11	0.34180 (9)	0.6897 (4)	0.57058 (10)	0.0190 (6)	
C14	0.31541 (11)	0.4229 (4)	0.55193 (13)	0.0219 (7)	
H14	0.2959	0.3242	0.5379	0.026*	
C15	0.35916 (11)	0.4038 (5)	0.58445 (14)	0.0246 (7)	
H15	0.3746	0.2931	0.5964	0.030*	
C16	0.37506 (11)	0.5770 (4)	0.59556 (13)	0.0220 (7)	
H16	0.4040	0.6112	0.6168	0.026*	
N12	0.26851 (9)	0.9206 (4)	0.56604 (11)	0.0207 (6)	
N13	0.31151 (9)	0.9582 (3)	0.59102 (11)	0.0187 (6)	
C17	0.31975 (12)	1.0505 (5)	0.63671 (14)	0.0256 (7)	
H17	0.3473	1.0900	0.6611	0.031*	
C18	0.28065 (13)	1.0760 (5)	0.64097 (15)	0.0331 (9)	
H18	0.2755	1.1379	0.6686	0.040*	
C19	0.24998 (12)	0.9929 (5)	0.59665 (14)	0.0262 (7)	
H19	0.2198	0.9885	0.5893	0.031*	
N14	0.20875 (8)	0.5902 (4)	0.52268 (11)	0.0195 (6)	
N15	0.16941 (9)	0.5474 (4)	0.48424 (11)	0.0188 (6)	
C20	0.20858 (11)	0.5150 (4)	0.56683 (13)	0.0220 (7)	
H20	0.2319	0.5215	0.6004	0.026*	
C21	0.16970 (12)	0.4255 (5)	0.55738 (14)	0.0262 (7)	
H21	0.1618	0.3611	0.5826	0.031*	
C22	0.14512 (11)	0.4486 (4)	0.50472 (13)	0.0217 (7)	
H22	0.1166	0.4044	0.4860	0.026*	
C23	0.15845 (11)	0.6126 (4)	0.43080 (13)	0.0188 (6)	
H23	0.1287	0.5681	0.4084	0.023*	
O1S	0.06346 (9)	0.4684 (4)	0.38493 (13)	0.0447 (7)	
H1S	0.0533	0.3683	0.3705	0.067*	
C1S	0.03005 (16)	0.5722 (8)	0.3923 (2)	0.0562 (13)	
H1S1	0.0428	0.6501	0.4237	0.084*	
H1S2	0.0090	0.4892	0.3974	0.084*	
H1S3	0.0155	0.6484	0.3610	0.084*	

### Atomic displacement parameters (Å^2^)

**Table:**

	*U*^11^	*U*^22^	*U*^33^	*U*^12^	*U*^13^	*U*^23^
Ni1	0.0240 (3)	0.0130 (3)	0.0281 (3)	−0.0013 (2)	0.0112 (2)	0.0001 (2)
C1	0.0249 (17)	0.0149 (16)	0.0311 (18)	−0.0032 (13)	0.0129 (14)	−0.0018 (13)
N1	0.0254 (15)	0.0134 (13)	0.0269 (14)	0.0003 (11)	0.0123 (12)	0.0014 (11)
N2	0.0199 (14)	0.0145 (13)	0.0261 (14)	−0.0017 (10)	0.0107 (11)	−0.0015 (11)
C2	0.0239 (17)	0.0188 (16)	0.0255 (17)	0.0024 (13)	0.0114 (14)	0.0016 (13)
C3	0.0216 (17)	0.0243 (17)	0.0278 (17)	−0.0021 (14)	0.0126 (14)	0.0007 (14)
C4	0.0239 (17)	0.0174 (16)	0.0258 (16)	−0.0037 (13)	0.0128 (14)	−0.0021 (13)
N3	0.0241 (15)	0.0135 (13)	0.0336 (16)	−0.0005 (11)	0.0121 (12)	−0.0009 (11)
N4	0.0217 (14)	0.0152 (13)	0.0290 (15)	−0.0009 (11)	0.0093 (12)	−0.0010 (11)
C5	0.0193 (17)	0.0281 (18)	0.0273 (18)	−0.0020 (14)	0.0075 (14)	−0.0043 (14)
C6	0.0223 (18)	0.037 (2)	0.0326 (19)	−0.0025 (15)	0.0053 (15)	0.0040 (16)
C7	0.0219 (17)	0.0202 (17)	0.041 (2)	−0.0002 (14)	0.0079 (15)	0.0092 (15)
N5	0.0266 (15)	0.0175 (14)	0.0324 (16)	−0.0019 (11)	0.0158 (13)	0.0000 (12)
N6	0.0241 (15)	0.0153 (13)	0.0343 (16)	−0.0006 (11)	0.0176 (12)	−0.0031 (11)
C8	0.0275 (19)	0.0284 (19)	0.0330 (19)	−0.0053 (15)	0.0151 (15)	0.0003 (15)
C9	0.0298 (19)	0.036 (2)	0.037 (2)	−0.0051 (16)	0.0198 (16)	−0.0094 (17)
C10	0.0249 (18)	0.0234 (18)	0.040 (2)	−0.0006 (14)	0.0153 (16)	−0.0080 (15)
N7	0.0352 (17)	0.0172 (14)	0.0327 (17)	0.0006 (12)	0.0141 (13)	−0.0011 (12)
C11	0.0242 (18)	0.0132 (16)	0.041 (2)	−0.0018 (13)	0.0157 (15)	−0.0005 (14)
S1	0.0471 (6)	0.0277 (5)	0.0401 (5)	−0.0074 (4)	0.0290 (5)	−0.0065 (4)
N8	0.0303 (16)	0.0204 (15)	0.0350 (17)	−0.0018 (12)	0.0157 (13)	0.0014 (13)
C12	0.0238 (17)	0.0198 (17)	0.0264 (17)	−0.0048 (14)	0.0138 (14)	−0.0030 (14)
S2	0.0291 (5)	0.0257 (4)	0.0254 (4)	−0.0008 (3)	0.0104 (3)	0.0054 (3)
N9	0.0317 (18)	0.0188 (16)	0.057 (2)	−0.0024 (13)	0.0135 (16)	0.0059 (14)
C13	0.040 (3)	0.019 (2)	0.042 (3)	−0.003 (2)	0.013 (2)	0.002 (2)
S3	0.0371 (10)	0.0350 (10)	0.0990 (16)	−0.0052 (7)	0.0315 (10)	0.0063 (9)
N9'	0.0317 (18)	0.0188 (16)	0.057 (2)	−0.0024 (13)	0.0135 (16)	0.0059 (14)
C13'	0.040 (3)	0.019 (2)	0.042 (3)	−0.003 (2)	0.013 (2)	0.002 (2)
S3'	0.0323 (18)	0.056 (2)	0.077 (3)	−0.0159 (15)	−0.0082 (16)	0.0269 (19)
Ni2	0.0191 (4)	0.0123 (4)	0.0213 (4)	0.0001 (3)	0.0100 (3)	0.0002 (3)
N10	0.0208 (14)	0.0133 (13)	0.0237 (14)	−0.0007 (10)	0.0105 (11)	−0.0002 (10)
N11	0.0226 (14)	0.0145 (13)	0.0213 (13)	−0.0015 (11)	0.0099 (11)	0.0003 (10)
C14	0.0272 (18)	0.0106 (15)	0.0311 (18)	0.0003 (13)	0.0147 (14)	0.0007 (13)
C15	0.0263 (18)	0.0159 (16)	0.0324 (18)	0.0057 (13)	0.0121 (15)	0.0019 (13)
C16	0.0200 (17)	0.0206 (17)	0.0256 (17)	0.0019 (13)	0.0089 (13)	0.0049 (13)
N12	0.0208 (14)	0.0164 (13)	0.0268 (15)	−0.0007 (11)	0.0112 (12)	−0.0019 (11)
N13	0.0212 (14)	0.0128 (13)	0.0237 (14)	0.0002 (10)	0.0105 (11)	−0.0009 (10)
C17	0.0332 (19)	0.0195 (17)	0.0246 (17)	−0.0043 (14)	0.0117 (15)	−0.0058 (13)
C18	0.044 (2)	0.0280 (19)	0.035 (2)	−0.0018 (16)	0.0233 (18)	−0.0105 (16)
C19	0.0296 (19)	0.0216 (17)	0.0336 (19)	0.0016 (14)	0.0190 (15)	−0.0036 (14)
N14	0.0184 (14)	0.0172 (13)	0.0223 (14)	0.0001 (11)	0.0071 (11)	0.0009 (11)
N15	0.0202 (14)	0.0151 (13)	0.0227 (14)	−0.0019 (10)	0.0101 (11)	0.0007 (10)
C20	0.0254 (18)	0.0208 (17)	0.0228 (16)	0.0017 (13)	0.0125 (14)	0.0017 (13)
C21	0.0312 (19)	0.0238 (18)	0.0287 (18)	−0.0015 (14)	0.0171 (15)	0.0044 (14)
C22	0.0242 (17)	0.0164 (16)	0.0278 (17)	−0.0034 (13)	0.0134 (14)	0.0024 (13)
C23	0.0230 (16)	0.0135 (15)	0.0219 (16)	0.0000 (12)	0.0109 (13)	0.0009 (12)
O1S	0.0299 (15)	0.0500 (19)	0.0543 (19)	−0.0059 (13)	0.0164 (13)	−0.0060 (15)
C1S	0.049 (3)	0.067 (3)	0.061 (3)	0.001 (2)	0.031 (2)	0.005 (3)

### Geometric parameters (Å, °)

**Table:**

Ni1—N8	2.046 (3)	Ni2—N14^i^	2.077 (3)
Ni1—N7	2.058 (3)	Ni2—N14	2.077 (3)
Ni1—N9	2.070 (3)	Ni2—N12^i^	2.081 (3)
Ni1—N1	2.080 (3)	Ni2—N12	2.081 (3)
Ni1—N5	2.097 (3)	Ni2—N10^i^	2.082 (3)
Ni1—N3	2.119 (3)	Ni2—N10	2.082 (3)
C1—N6	1.444 (4)	N10—C14	1.325 (4)
C1—N4	1.447 (4)	N10—N11	1.355 (4)
C1—N2	1.450 (4)	N11—C16	1.342 (4)
C1—H1	1.0000	N11—C23^i^	1.449 (4)
N1—C2	1.317 (4)	C14—C15	1.396 (5)
N1—N2	1.358 (4)	C14—H14	0.9500
N2—C4	1.352 (4)	C15—C16	1.365 (5)
C2—C3	1.402 (5)	C15—H15	0.9500
C2—H2	0.9500	C16—H16	0.9500
C3—C4	1.369 (5)	N12—C19	1.327 (4)
C3—H3	0.9500	N12—N13	1.362 (4)
C4—H4	0.9500	N13—C17	1.349 (4)
N3—C5	1.322 (5)	N13—C23^i^	1.447 (4)
N3—N4	1.364 (4)	C17—C18	1.369 (5)
N4—C7	1.355 (5)	C17—H17	0.9500
C5—C6	1.405 (5)	C18—C19	1.390 (5)
C5—H5	0.9500	C18—H18	0.9500
C6—C7	1.365 (6)	C19—H19	0.9500
C6—H6	0.9500	N14—C20	1.326 (4)
C7—H7	0.9500	N14—N15	1.367 (4)
N5—C8	1.328 (5)	N15—C22	1.358 (4)
N5—N6	1.355 (4)	N15—C23	1.441 (4)
N6—C10	1.359 (4)	C20—C21	1.389 (5)
C8—C9	1.394 (5)	C20—H20	0.9500
C8—H8	0.9500	C21—C22	1.364 (5)
C9—C10	1.369 (5)	C21—H21	0.9500
C9—H9	0.9500	C22—H22	0.9500
C10—H10	0.9500	C23—N13^i^	1.447 (4)
N7—C11	1.160 (5)	C23—N11^i^	1.449 (4)
C11—S1	1.627 (4)	C23—H23	1.0000
N8—C12	1.160 (5)	O1S—C1S	1.429 (6)
C12—S2	1.629 (4)	O1S—H1S	0.8400
N9—C13	1.237 (7)	C1S—H1S1	0.9800
C13—S3	1.652 (7)	C1S—H1S2	0.9800
C13'—S3'	1.713 (12)	C1S—H1S3	0.9800
			
N8—Ni1—N7	92.74 (12)	N14^i^—Ni2—N12^i^	95.27 (10)
N8—Ni1—N9	92.42 (13)	N14—Ni2—N12^i^	84.73 (10)
N7—Ni1—N9	92.02 (14)	N14^i^—Ni2—N12	84.73 (10)
N8—Ni1—N1	93.31 (12)	N14—Ni2—N12	95.27 (10)
N7—Ni1—N1	91.68 (12)	N12^i^—Ni2—N12	179.996 (1)
N9—Ni1—N1	173.02 (12)	N14^i^—Ni2—N10^i^	93.96 (10)
N8—Ni1—N5	93.02 (12)	N14—Ni2—N10^i^	86.04 (10)
N7—Ni1—N5	173.64 (12)	N12^i^—Ni2—N10^i^	85.81 (10)
N9—Ni1—N5	90.41 (13)	N12—Ni2—N10^i^	94.19 (10)
N1—Ni1—N5	85.31 (11)	N14^i^—Ni2—N10	86.04 (10)
N8—Ni1—N3	175.06 (12)	N14—Ni2—N10	93.96 (10)
N7—Ni1—N3	89.98 (12)	N12^i^—Ni2—N10	94.19 (10)
N9—Ni1—N3	91.61 (12)	N12—Ni2—N10	85.81 (10)
N1—Ni1—N3	82.48 (11)	N10^i^—Ni2—N10	180.00 (15)
N5—Ni1—N3	84.08 (11)	C14—N10—N11	104.9 (3)
N6—C1—N4	109.7 (3)	C14—N10—Ni2	137.4 (2)
N6—C1—N2	110.8 (3)	N11—N10—Ni2	117.63 (19)
N4—C1—N2	109.4 (3)	C16—N11—N10	112.2 (3)
N6—C1—H1	109.0	C16—N11—C23^i^	128.6 (3)
N4—C1—H1	109.0	N10—N11—C23^i^	119.3 (3)
N2—C1—H1	109.0	N10—C14—C15	110.7 (3)
C2—N1—N2	105.4 (3)	N10—C14—H14	124.6
C2—N1—Ni1	135.1 (2)	C15—C14—H14	124.6
N2—N1—Ni1	119.2 (2)	C16—C15—C14	105.8 (3)
C4—N2—N1	111.6 (3)	C16—C15—H15	127.1
C4—N2—C1	128.5 (3)	C14—C15—H15	127.1
N1—N2—C1	119.6 (3)	N11—C16—C15	106.4 (3)
N1—C2—C3	111.0 (3)	N11—C16—H16	126.8
N1—C2—H2	124.5	C15—C16—H16	126.8
C3—C2—H2	124.5	C19—N12—N13	105.2 (3)
C4—C3—C2	105.5 (3)	C19—N12—Ni2	136.8 (2)
C4—C3—H3	127.2	N13—N12—Ni2	117.43 (19)
C2—C3—H3	127.2	C17—N13—N12	111.4 (3)
N2—C4—C3	106.5 (3)	C17—N13—C23^i^	129.2 (3)
N2—C4—H4	126.8	N12—N13—C23^i^	119.2 (3)
C3—C4—H4	126.8	N13—C17—C18	106.5 (3)
C5—N3—N4	105.3 (3)	N13—C17—H17	126.8
C5—N3—Ni1	136.5 (2)	C18—C17—H17	126.8
N4—N3—Ni1	118.1 (2)	C17—C18—C19	106.0 (3)
C7—N4—N3	111.3 (3)	C17—C18—H18	127.0
C7—N4—C1	128.7 (3)	C19—C18—H18	127.0
N3—N4—C1	119.6 (3)	N12—C19—C18	110.8 (3)
N3—C5—C6	111.0 (3)	N12—C19—H19	124.6
N3—C5—H5	124.5	C18—C19—H19	124.6
C6—C5—H5	124.5	C20—N14—N15	105.2 (3)
C7—C6—C5	105.4 (3)	C20—N14—Ni2	137.7 (2)
C7—C6—H6	127.3	N15—N14—Ni2	117.06 (19)
C5—C6—H6	127.3	C22—N15—N14	111.0 (3)
N4—C7—C6	107.0 (3)	C22—N15—C23	129.3 (3)
N4—C7—H7	126.5	N14—N15—C23	119.7 (3)
C6—C7—H7	126.5	N14—C20—C21	111.0 (3)
C8—N5—N6	105.1 (3)	N14—C20—H20	124.5
C8—N5—Ni1	136.0 (2)	C21—C20—H20	124.5
N6—N5—Ni1	118.8 (2)	C22—C21—C20	106.2 (3)
N5—N6—C10	111.5 (3)	C22—C21—H21	126.9
N5—N6—C1	119.8 (3)	C20—C21—H21	126.9
C10—N6—C1	128.5 (3)	N15—C22—C21	106.6 (3)
N5—C8—C9	111.2 (3)	N15—C22—H22	126.7
N5—C8—H8	124.4	C21—C22—H22	126.7
C9—C8—H8	124.4	N15—C23—N13^i^	110.5 (3)
C10—C9—C8	105.5 (3)	N15—C23—N11^i^	110.8 (3)
C10—C9—H9	127.2	N13^i^—C23—N11^i^	110.3 (3)
C8—C9—H9	127.2	N15—C23—H23	108.4
N6—C10—C9	106.6 (3)	N13^i^—C23—H23	108.4
N6—C10—H10	126.7	N11^i^—C23—H23	108.4
C9—C10—H10	126.7	C1S—O1S—H1S	109.5
C11—N7—Ni1	147.0 (3)	O1S—C1S—H1S1	109.5
N7—C11—S1	177.7 (3)	O1S—C1S—H1S2	109.5
C12—N8—Ni1	170.1 (3)	H1S1—C1S—H1S2	109.5
N8—C12—S2	177.7 (3)	O1S—C1S—H1S3	109.5
C13—N9—Ni1	144.9 (4)	H1S1—C1S—H1S3	109.5
N9—C13—S3	178.5 (5)	H1S2—C1S—H1S3	109.5
N14^i^—Ni2—N14	180.00 (10)		
			
N8—Ni1—N1—C2	−54.5 (3)	N8—Ni1—N7—C11	−174.3 (5)
N7—Ni1—N1—C2	38.3 (3)	N9—Ni1—N7—C11	−81.8 (5)
N5—Ni1—N1—C2	−147.3 (3)	N1—Ni1—N7—C11	92.3 (5)
N3—Ni1—N1—C2	128.1 (3)	N3—Ni1—N7—C11	9.8 (5)
N8—Ni1—N1—N2	133.0 (2)	N8—Ni1—N9—C13	−83.5 (6)
N7—Ni1—N1—N2	−134.1 (2)	N7—Ni1—N9—C13	−176.4 (6)
N5—Ni1—N1—N2	40.2 (2)	N5—Ni1—N9—C13	9.5 (6)
N3—Ni1—N1—N2	−44.4 (2)	N3—Ni1—N9—C13	93.6 (6)
C2—N1—N2—C4	−0.2 (4)	N14^i^—Ni2—N10—C14	−135.9 (3)
Ni1—N1—N2—C4	174.3 (2)	N14—Ni2—N10—C14	44.1 (3)
C2—N1—N2—C1	−173.8 (3)	N12^i^—Ni2—N10—C14	−40.9 (3)
Ni1—N1—N2—C1	0.7 (4)	N12—Ni2—N10—C14	139.1 (3)
N6—C1—N2—C4	127.4 (3)	N14^i^—Ni2—N10—N11	41.9 (2)
N4—C1—N2—C4	−111.5 (4)	N14—Ni2—N10—N11	−138.1 (2)
N6—C1—N2—N1	−60.1 (4)	N12^i^—Ni2—N10—N11	136.9 (2)
N4—C1—N2—N1	61.0 (4)	N12—Ni2—N10—N11	−43.1 (2)
N2—N1—C2—C3	−0.3 (4)	C14—N10—N11—C16	0.3 (3)
Ni1—N1—C2—C3	−173.5 (2)	Ni2—N10—N11—C16	−178.1 (2)
N1—C2—C3—C4	0.6 (4)	C14—N10—N11—C23^i^	179.5 (3)
N1—N2—C4—C3	0.5 (4)	Ni2—N10—N11—C23^i^	1.0 (3)
C1—N2—C4—C3	173.5 (3)	N11—N10—C14—C15	−0.2 (4)
C2—C3—C4—N2	−0.7 (4)	Ni2—N10—C14—C15	177.8 (2)
N7—Ni1—N3—C5	−38.7 (3)	N10—C14—C15—C16	0.0 (4)
N9—Ni1—N3—C5	53.3 (4)	N10—N11—C16—C15	−0.3 (4)
N1—Ni1—N3—C5	−130.4 (3)	C23^i^—N11—C16—C15	−179.4 (3)
N5—Ni1—N3—C5	143.5 (3)	C14—C15—C16—N11	0.1 (4)
N7—Ni1—N3—N4	137.9 (2)	N14^i^—Ni2—N12—C19	144.3 (4)
N9—Ni1—N3—N4	−130.0 (2)	N14—Ni2—N12—C19	−35.7 (4)
N1—Ni1—N3—N4	46.2 (2)	N10^i^—Ni2—N12—C19	50.7 (4)
N5—Ni1—N3—N4	−39.8 (2)	N10—Ni2—N12—C19	−129.3 (4)
C5—N3—N4—C7	0.0 (4)	N14^i^—Ni2—N12—N13	−45.7 (2)
Ni1—N3—N4—C7	−177.6 (2)	N14—Ni2—N12—N13	134.3 (2)
C5—N3—N4—C1	173.4 (3)	N10^i^—Ni2—N12—N13	−139.3 (2)
Ni1—N3—N4—C1	−4.3 (4)	N10—Ni2—N12—N13	40.7 (2)
N6—C1—N4—C7	−124.3 (4)	C19—N12—N13—C17	0.7 (4)
N2—C1—N4—C7	114.0 (4)	Ni2—N12—N13—C17	−172.2 (2)
N6—C1—N4—N3	63.7 (4)	C19—N12—N13—C23^i^	176.2 (3)
N2—C1—N4—N3	−58.1 (4)	Ni2—N12—N13—C23^i^	3.3 (3)
N4—N3—C5—C6	0.5 (4)	N12—N13—C17—C18	−1.0 (4)
Ni1—N3—C5—C6	177.4 (3)	C23^i^—N13—C17—C18	−176.0 (3)
N3—C5—C6—C7	−0.8 (4)	N13—C17—C18—C19	0.9 (4)
N3—N4—C7—C6	−0.5 (4)	N13—N12—C19—C18	−0.1 (4)
C1—N4—C7—C6	−173.1 (3)	Ni2—N12—C19—C18	170.7 (3)
C5—C6—C7—N4	0.8 (4)	C17—C18—C19—N12	−0.5 (4)
N8—Ni1—N5—C8	50.3 (4)	N12^i^—Ni2—N14—C20	138.3 (3)
N9—Ni1—N5—C8	−42.1 (4)	N12—Ni2—N14—C20	−41.7 (3)
N1—Ni1—N5—C8	143.4 (4)	N10^i^—Ni2—N14—C20	−135.6 (3)
N3—Ni1—N5—C8	−133.7 (4)	N10—Ni2—N14—C20	44.4 (3)
N8—Ni1—N5—N6	−132.6 (3)	N12^i^—Ni2—N14—N15	−43.9 (2)
N9—Ni1—N5—N6	135.0 (3)	N12—Ni2—N14—N15	136.1 (2)
N1—Ni1—N5—N6	−39.5 (2)	N10^i^—Ni2—N14—N15	42.2 (2)
N3—Ni1—N5—N6	43.4 (2)	N10—Ni2—N14—N15	−137.8 (2)
C8—N5—N6—C10	0.3 (4)	C20—N14—N15—C22	0.7 (3)
Ni1—N5—N6—C10	−177.6 (2)	Ni2—N14—N15—C22	−177.7 (2)
C8—N5—N6—C1	175.6 (3)	C20—N14—N15—C23	178.6 (3)
Ni1—N5—N6—C1	−2.3 (4)	Ni2—N14—N15—C23	0.2 (3)
N4—C1—N6—N5	−59.9 (4)	N15—N14—C20—C21	−0.3 (4)
N2—C1—N6—N5	60.9 (4)	Ni2—N14—C20—C21	177.6 (3)
N4—C1—N6—C10	114.5 (4)	N14—C20—C21—C22	−0.2 (4)
N2—C1—N6—C10	−124.6 (4)	N14—N15—C22—C21	−0.8 (4)
N6—N5—C8—C9	−0.1 (4)	C23—N15—C22—C21	−178.5 (3)
Ni1—N5—C8—C9	177.3 (3)	C20—C21—C22—N15	0.6 (4)
N5—C8—C9—C10	−0.1 (4)	C22—N15—C23—N13^i^	−121.3 (3)
N5—N6—C10—C9	−0.4 (4)	N14—N15—C23—N13^i^	61.3 (4)
C1—N6—C10—C9	−175.1 (3)	C22—N15—C23—N11^i^	116.3 (4)
C8—C9—C10—N6	0.3 (4)	N14—N15—C23—N11^i^	−61.2 (4)

Symmetry codes: (i) −*x*+1/2, −*y*+3/2, −*z*+1.

### Hydrogen-bond geometry (Å, °)

**Table:**

*D*—H···*A*	*D*—H	H···*A*	*D*···*A*	*D*—H···*A*
O1S—H1S···S3^ii^	0.84	2.43	3.267 (4)	175.
O1S—H1S···S3'^ii^	0.84	2.88	3.459 (6)	128.
C23—H23···O1S	1.00	2.15	3.118 (4)	162.

Symmetry codes: (ii) *x*−1/2, −*y*+1/2, *z*−1/2.
